# Prevalence of postprandial hyperglycaemia in basal insulin-treated patients with type 2 diabetes mellitus with controlled fasting glycaemia and elevated glycosylated haemoglobin

**DOI:** 10.1186/s13098-019-0452-8

**Published:** 2019-07-24

**Authors:** Francisco J. Tinahones, María Molina-Vega, Juan Parra-Barona, Juana Flores-Le Roux, Ricardo Gómez-Huelgas

**Affiliations:** 1 Hospital Universitario Virgen de la Victoria, Servicio de Endocrinología, Campus Universitario de Teatinos s/n., 29010 Málaga, Spain; 2 CIBER de Fisiopatología de la Obesidad y Nutrición (CIBEROBN), Instituto de Salud Carlos III (ISCIII), Málaga, Spain; 3 Hospital de Mérida, Mérida, Spain; 4 Hospital del Mar, Barcelona, Spain; 5 Hospital Universitario Regional, Servicio de Medicina Interna, FIMABIS, Málaga, Spain

**Keywords:** Hyperglycaemia, Postprandial glucose, Type 2 diabetes mellitus, HbA1c

## Abstract

**Objective:**

To study the prevalence of postprandial hyperglycaemia (PPH) in type 2 diabetes mellitus (T2DM) patients treated with basal insulin, having fasting glucose < 130 mg/dL but HbA1c > 7.0% (53 mmol/mol).

**Methods:**

This was an observational prospective multicentric study conducted in Spain. During 2 weeks, patients recorded a 6-point self-measured blood glucose profile (before and 2 h after eating) every 2 days. PPH was defined according to IDF and ADA guidelines (> 160 and > 180 mg/dL, respectively).

**Results:**

We included 98 patients (males: 56.1%; mean age: 64.3 ± 10.4 years) who were treated with basal insulin for at least 1 year at stable doses in the last 2 months, 88.8% of them received concomitant oral antidiabetic drugs. Overall, 95.7% (95% CI 91.6–99.8) and 93.5% (95% CI 88.6–98.5) of patients showed ≥ 1 episode of PPH according to IDF and ADA criteria respectively. PPH was more frequently observed after lunch and dinner. The proportion of patients with ≥ 40% readings in range of PPH was 59.1% (95% CI 49.1–69.1) and 40.9% (95% CI 30.9–50.9), according to IDF and ADA criteria, respectively.

**Conclusions:**

PPH is very common and should be considered a priority target in basal insulin-treated T2DM patients with elevated HbA1c despite controlled fasting glucose.

## Introduction

Diabetes is one of the most important public health challenges worldwide. Its related clinical complications have doubled the prevalence over the past three decades [1] and it is estimated that 10.1% of worldwide adult population will suffer diabetes by 2035 [2].

The glycosylated haemoglobin (HbA1c), is the most accepted measure of chronic glycaemic levels and the most accepted target of diabetic control since it was recommended in the routinely follow-up, due to its good correlation with the risk of diabetic complications [3]. Fasting glycaemia (FG), postprandial glycaemia (PPG) and average plasmatic glycaemia are strongly correlated with HbA1c [4, 5].

It has been shown that postprandial hyperglycaemia (PPH) is common among people with type 1 and type 2 diabetes [6, 7] even if considered controlled according to their levels of HbA1c [8]. PPH has been defined as a postprandial glucose > 180 mg/dL by the American Diabetes Association (ADA) or > 160 mg/dL by the International Diabetes Federation (IDF)0 [9, 10]. Epidemiological studies have shown an association between PPG levels and the development of diabetic complications and cardiovascular risk [11–14]. It seems clear that PPG contributes to HbA1c levels and that this contribution is even greater than that from FG when HbA1c is closer to 7.0% (53 mmol/mol). A study using continuous glucose monitoring in patients with T2DM showed that FG was very similar between the groups with HbA1c < 6.5% (< 48 mmol/mol), 6.5% to < 7.0% (48 mmol/mol to < 53 mmol/mol), and 7.0% to < 8.0% (53 mmol/mol to < 64 mmol/mol), being the main difference the PPG [15]. These data suggest that reduction of HbA1c in patients who are closer to target will be best achieved if the therapeutic target is the control of PPG levels [9, 15, 16].

Prevalence of PPH is unknown in Spanish population. This study aims to estimate the prevalence of PPH in Spain among T2DM patients treated with basal insulin who present controlled levels of FG. This knowledge could help to identify the patient‘s profile that could benefit from optimized therapeutic management.

## Subjects and methods

### Subjects

In this study, the participating investigators are from different autonomous communities of Spain. All patients were included in the study from April 2013 till July 2014. The first patients attended by every investigator, meeting all the selection criteria, were included in the study once they have their writing informed consent. Male and female outpatients aged 40 or older diagnosed with T2DM at least 1 year ago were eligible for this study. Patients must have controlled fasting glycaemia (FG < 130 mg/dL [7.2 mmol/L]) [17] and uncontrolled HbA1c (7.0% to 9.0% [53 to 75 mmol/mol]). They must be treated with basal insulin during at least 1 year before inclusion and at stable doses for the last 2 months (oscillations of up to ± 20% were allowed). Concomitant use of 1 to 3 oral antidiabetic drugs (OAD) (metformin, sulfonylureas, or dipeptidyl peptidase-4 inhibitors) at stable doses was allowed per protocol. Serious comorbidities, use of systemic corticosteroids and people with type 1 diabetes were excluded.

Patients must be able to comply with the study procedures. All included subjects gave their informed consent to participation prior to inclusion.

### Design and methodology

This observational, prospective, multicentre study was designed to estimate the prevalence of PPH according to the IDF and ADA guidelines, among treated T2DM patients with HbA1c > 7.0% (> 53 mmol/mol) despite having controlled FG (< 130 mg/dL).

The study included a basal visit (day 0) and a final visit (+14 days). The recruitment period encompassed 14 months. Data about diabetes characteristics, comorbidities and treatment profile were recorded basally and in the final visit. Patients were asked to record data about their 6-point glycaemic profile every 2 days over the 2 weeks follow-up period. Participants were asked to register a 6-point self-measured blood glucose levels before and after each meal (breakfast, lunch and dinner). Each participant received a glucometer BGStar^®^ which should be returned to their physician at the end of the study. Diet composition and physical activity must have been stable during 3 months before inclusion and during the study, in order to avoid any changes and potential confusing factors.

The study was carried out in 16 centers (hospitals and specialized endocrinology clinics) across Spain. It complied with ethical standards according to International Conference of Harmonization (ICH) and was approved by Ethics Committees of participating centers in accordance with local and national regulations.

### Statistics

All patients fulfilling selection criteria stated in the protocol were considered for analysis. Descriptive statistical analyses were primarily performed. Continuous variables were described in terms of number of patients with valid observations, mean, standard deviation (SD), median, interquartile range (IQR). Categorical variables were described by frequencies and corresponding 95% confidence intervals (CI).

Continuous variables were assessed for normal distribution using the Kolmogorov- Smirnov test. Variables following the normal distribution were evaluated using the Student *t* test or ANOVA. When data distribution was non-normal, non-parametric tests such as Mann–Whitney U or Kruskal–Wallis were applied. For categorical variables, according to the distribution of the variable in the response category, the Chi square or the exact Fisher test was applied when necessary. Two-tailed α-error of 0.05 was applied in statistical tests. Statistical analysis was performed using SAS^®^ statistical software version 9.4.

## Results

A total of 108 patients were included and 98 were valid for study analyses (43 [43.9%] females and 55 [56.1%] males). Mean (SD) age was 64.3 ± 10.4 years. Description of the study sample is summarized in Table 1. There were no changes in diet or physical activity during the study.

**Table 1:** Patient descriptive characteristics

Patients’ physical exploration		
N = 98	Basal visit	Final visit
Weight (kg)	82.2 (16.0)	81.8 (15.9)
Body mass index (BMI) (kg/m^2^)	30.0 (5.2)	29.8 (5.2)
Waist perimeter (WP) (cm)	101.3 (13.1)	102.2 (14.8)
Abdominal obesity		
According to IDF (WP > 80 cm women and > 90 cm men)	89 (90.8%)	88 (89.8%)
According to GLESMO (WP > 88 cm women and > 94 cm men)	76 (77.6%)	78 (79.6%)
According to NCEP-ATP III (WP > 88 cm women and > 102 cm men)	62 (63.3%)	62 (63.3%)
Fasting glycaemia (mg/dL)	107.7 (16.8)	
HbA1c (%)	7.9 (0.6)	
HbA1c (mmol/mol)	63 (6)	
Oral antidiabetic drugs used		
Basal insulin dose (U/day)	31.6 (17)	31.6 (16.9)
Any OAD	87 (88.8%)	89 (90.8%)
Metformin	80 (81.6%)	81 (82.7%)
Sulfonylureas	31 (31.6%)	31 (31.6%)
DPP-4 inhibitor	30 (30.6%)	31 (31.6%)

Data are presented as mean (SD) or n (%). *AMI* acute myocardial infarction, *ALO* acute lung oedema, *OAD* oral antidiabetic drug

Regarding the T2DM characteristics, mean (SD) HbA1c was 7.9 ± 0.6% (63 ± 6 mmol/mol) and mean (SD) FG was 107.7 ± 16.8 mg/dL. Patients had a mean (SD) of 16.4 ± 8.5 years since diagnosis and 77.6% patients had been diagnosed more than 10 years ago. Diabetes-related complications were present in 61.2% patients. Retinopathy was the most frequent complication observed (32.7% patients), followed by other cardiovascular complications (25.5%) like acute myocardial infarction, angina, hospitalization due to heart failure, stroke, acute lung oedema or revascularization. Nephropathy was present in 24.5% patients and neuropathy in 10.2%. Comorbidities most commonly observed were dyslipidemia (64.3% patients) and hypertension (64.3%).

Analysed patients were treated with basal insulin for a median (P25/P75) of 4.4 (2.1/7.5) years prior to inclusion and the current dosage remained unchanged a median (P25/P75) of 11.9 (6.3/18.0) months before inclusion. Insulin dose median (P25/P75) in the basal visit was 28.0 (18.0/38.0) units per day and it remained unchanged over the study. A high percentage of patients (88.8%) added any oral antidiabetic drugs (OAD) to their treatment (Table 1). Around half of them (50.6%) received metformin combined with sulfonylureas, DPP4 inhibitors or both. Mean OAD treatment duration was 9.9 ± 6.9 years (metformin), 10.4 ± 7.3 years (sulfonylureas) and 2.4 ± 1.7 years (DPP4 inhibitors).

### Main analysis

PPG was calculated in 93 patients who had this data available. A total of 55 patients (59.1%; CI 95% 49.1–69.1) had at least 40% postprandial glucose measurements above 160 mg/dL, and 38 patients (40.9%; CI 95% 30.9–50.9) had at least 40% PPG measurements above 180 mg/dL.

**Fig. 1:**
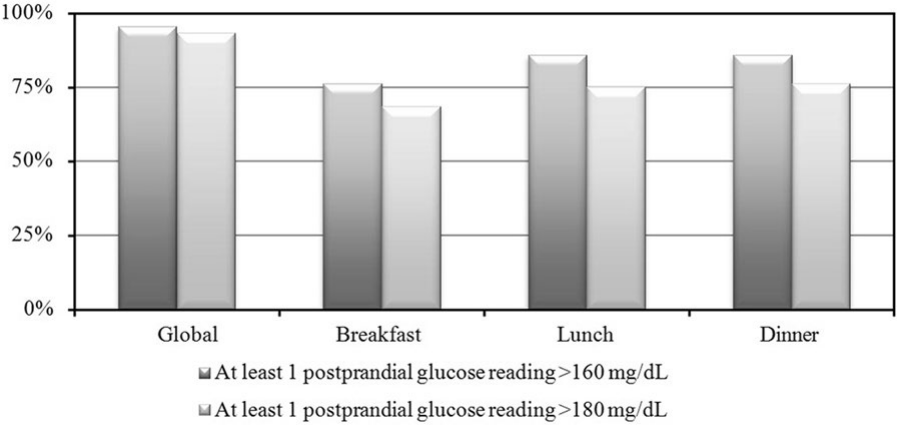
Percentage of patients with at least 1 measurement of postprandial hyperglycaemia during the study

Mean (CI 95%) PPG was 167.1 (159.4–174.9) mg/dL and almost all patients had at least one PPG measurement > 160 mg/dL (89; 95.7%) or > 180 mg/dL (87; 93.5%) (Fig. 1). The time of the day that showed more frequently elevated PPG levels was after lunch and dinner.

**Fig. 2:**
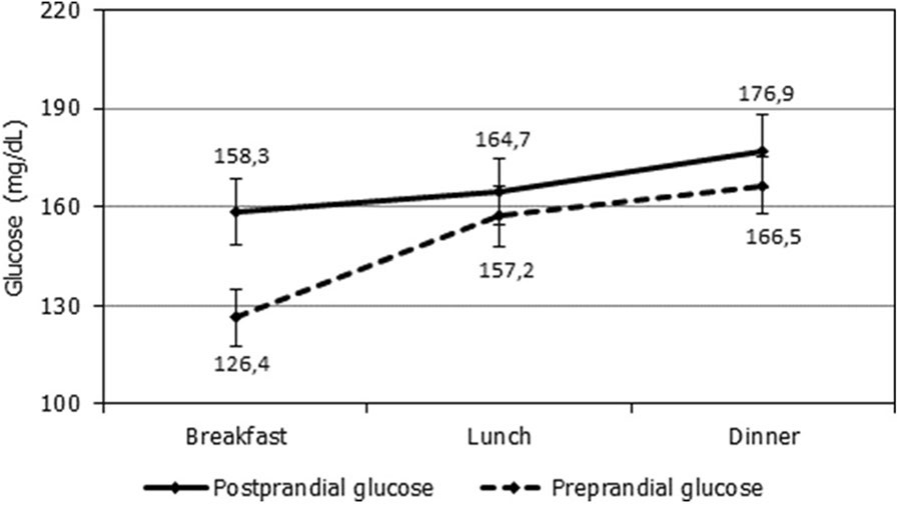
Average glucose measurements (mg/dL) during the study according to the reading time (mean values at each point showed)

The mean FG was 126.4 ± 32.1 mg/dL, while the mean of all preprandial glycaemia was 156.4 ± 36.9 mg/dL (Fig. 2).

Among patients with at least 40% PPG measurements > 160 mg/dL, 81.8% had more than 10 years since diagnosis, compared to 68.4% patients among those with < 40% PPG > 160 mg/dL (p = 0.03). There were more patients with at least 40% PPG > 160 mg/dL who received OAD monotherapy with metformin or had longer treatment with sulfonylureas. No other differences were observed (Table 2).

**Table 2:** Patients comparison based on the presence or absence of ≥ 40% PPG > 160 mg/dL

Patients with at least 40% PPG measurements > 160 mg/dL	No (N = 38)	Yes (N = 55)	P-value
Sex (male/female)	19/19 (50.0/50.0%)	33/19 (60.0/50.0%)	0.3982 (a)
Age (years)	65.0 (10.8)	64.0 (10.6)	0.6776 (b)
BMI (kg/m^2^)	30.1 (6.0)	29.7 (4.6)	0.7069 (b)
Systolic blood pressure (mmHg)	140.1 (16.3)	137.7 (15.2)	0.4653 (b)
Diastolic blood pressure (mmHg)	79.9 (9.5)	76.6 (8.8)	0.0885 (b)
Total cholesterol (mg/dL)	166.2 (32.5)	163.6 (30.8)	0.7034 (b)
Triglycerides (mg/dL)	148.0 (90.2)	153.7 (101.5)	0.7816 (b)
LDL cholesterol (mg/dL)	88.8 (34.1)	91.8 (27.8)	0.6394 (b)
HDL colesterol (mg/dL)	51.7 (19.6)	43.8 (11.5)	*0.0318 (b)*
Time since diagnosis (years)	14.5 (8.9)	17.3 (8.0)	0.1231 (b)
≤ 2 years	3 (7.9%)	0 (0.0%)	*0.0302 (c)*
2.1 to 5 years	3 (7.9%)	2 (3.6%)	
5.1 to 10 years	6 (15.8%)	8 (14.5%)	
> 10 years	26 (68.4%)	45 (81.8%)	
HbA1c (%)	7.8 (0.5)	8.0 (0.5)	0.0912 (b)
HbA1c (mmol/mol)	62 (5)	64 (5)	
Treatment with basal insulin: final dose (units/day)	31.1 (16.1)	31.1 (17.6)	0.9869 (a)
Years of treatment	5.0 (4.7)	5.6 (4.4)	0.5474 (b)
Treatment with ADO	33 (86.8%)	49 (89.1%)	0.7543 (a)
Years of treatment	9.2 (7.5)	11.7 (7.1)	0.1283 (b)
Treatment with metformin	30 (78.9%)	45 (81.8%)	0.7927 (a)
Years of treatment	9.8 (7.6)	10.4 (6.6)	0.7361 (b)
Treatment with DPP-4 Inhibitor	8 (21.1%)	20 (36.4%)	0.1673 (a)
Years of treatment	1.9 (1.5)	2.6 (1.8)	0.2983 (b)
Treatment with sulfonylurea	8 (21.1%)	21 (38.2%)	0.1110 (a)
Years of treatment	6.7 (4.9)	12.7 (7.2)	*0.0379 (b)*

Data are shown as n (%) or mean (SD). (a) Exact-Fisher, (b) independent T-student, (c) exact-Mantel–Haenszel

Italic values indicate statistically significant comparison

Patients with at least 40% PPG > 180 mg/dL, but not patients with ≥ 40% PPG > 160 mg/dL, showed a higher HbA1c (8.1 ± 0.5% vs. 7.8 ± 0.5% [65 ± 5 vs 62 ± 5 mmol/mol]; p = 0.007).

## Discussion

The purpose of this study was to estimate the frequency of PPG according to IDF [10] and ADA [9] guidelines in patients treated with basal insulin with control of FG but uncontrolled HbA1c (HbA1c > 7% [> 53 mmol/mol]). Describing clinical characteristics linked to patients with PPH was an additional aim.

Our results show that a nearly 60%/40% of treated T2DM patients have PPH (above 160/180 mg/dL) in at least 40% measurements, what means almost 1 out of 2 patients according to IDF criteria and more than 1 out of 3 of them according to ADA criteria. It is well-known that not only FG but also PPG contribute to HbA1c levels [9, 18] and that the triad FG, HbA1c and PPG is key for the adequate clinical control of T2DM [16, 19], though the role played by FG and PPG seems to be different. It has been shown that FG correlates better with the HbA1c level in patients with HbA1c value over 8.0%, while PPG appears to be strongly correlated with HbA1c level when this value is below 8.0% (64 mmol/mol) [20]. Our patients had a mean HbA1c of 7.9% (63 mmol/mol), so our data confirm the importance of PPG in the glycaemic control of patients with HbA1c fewer than 8% (64 mmol/mol).

PPH might play an important role in the development of diabetic microangiopathy [21] and may increase the risk of cardiovascular events [19, 22]. The large Diabetes Intervention Study [23], with an 11-year follow-up period, found that PPG was an independent risk factor for death. A more recent study [24] showed, after 14 years of follow-up, that both HbA1c and PPH are strong predictors for cardiovascular events and all-cause mortality in T2DM. In spite of this data, the relevance of targeting PPG in therapeutic schemes is still matter of some debate [25, 26], partially due to the results of the HEART2D trial [27]. This randomized controlled clinical trial comparing the effects of prandial versus fasting glycaemic control on risk for cardiovascular outcomes showed less than expected differences in PPG (0.8 mmol/L lower in prandial group, p < 0.01; however a difference of 2.5 mmol/L was initially presumed). Nevertheless, growing evidence points towards integration of PPG into daily diabetes control [28].

We also found that almost all patients presented at least one measurement > 160 mg/dL and/or > 180 mg/dL, with progressive increments of glycaemic values over the day. A recent work evaluating 500 patients with T2DM across different countries revealed that the highest PPG takes place after dinner, whereas the highest glucose increments occur after breakfast, pattern similar to that obtained in our study [29]. The awareness of this changing glucose pattern could be of importance for the appropriate management of these patients.

When the profile of patients with at least 40% PPH, according to ADA criteria, was studied, a significant difference of 0.3% higher HbA1c was found compared to those not showing such percentage.

Patients with at least 40% glucose readings > 160 mg/dL also showed to be treated with sulfonylureas for longer time. This feature is consistent with that obtained by Bonora et al. [6], who studied a large T2DM population and found that patients treated with sulfonylureas had greater postprandial glucose excursions that those patients not taking these drugs. These results consistently suggest that sulfonylureas do not have a preventive effect over PPH, so that patients with poor glycaemic control would benefit from alternative therapies with a higher impact in the PPH control.

Limitations to the interpretation of our results are related with the observational nature of the study. The recommendation provided by the study protocol was to self-monitor the PPG 2h after each meal following international guidelines [10, 17]. Some degree of heterogeneity regarding the time elapsed between each meal and glucose self-monitoring among participants may have over or underestimated glucose measurements. Nevertheless the high self-awareness and appropriate self-care of patients about the disease would minimize partially this variability.

In conclusion, PPH is a common feature in basal insulin-treated T2DM patients with elevated HbA1c despite controlled FG. Those patients with more PPH showed higher HbA1c, suggesting that control of FG is not enough and PPG should be also a therapeutic target to improve the diabetes control and to reduce the risk of diabetes related complications.

## Data Availability

The datasets used and/or analysed during the current study are available from the corresponding author on reasonable request.
